# Congenital radioulnar synostosis presenting in adulthood - a case report

**DOI:** 10.11604/pamj.2020.36.75.21413

**Published:** 2020-06-09

**Authors:** Mohammed Hamid Karrar Alsharif, Juman Mahmoud Almasaad, Khalid Mohammed Taha, Abubaker Yousif Elamin, Nagi Mahmoud Bakhit, Mohammed Ahammed Noureddin, Abair Awadalla Ahmed Mahdi

**Affiliations:** 1 Department of Basic Medical Science, College of Medicine, Prince Sattam Bin Abdulaziz University, Al Kharj, KSA,; 2 Department of Histology and Embryology, Medical Faculty, Ondokuz Mayis University, Samsun, Turkey,; 3 Department of Anatomy, Faculty of Medicine, National University, Khartoum, Sudan,; 4 College of Medicine, King Saud Bin Abdulaziz for Health Sciences University, Jeddah, KSA,; 5 Department of Anatomy, Faculty of Medicine, El Deain University, El Deain, Sudan,; 6 Department of Anatomy, Faculty of Medicine, University of Science and Technology, Khartoum, Sudan,; 7 Department of Medicine, College of Medicine, Prince Sattam Bin Abdulaziz University, Al Kharj, KSA,; 8 College of Medicine, King Saud Bin Abdulaziz for Health Sciences University, Riyadh, KSA

**Keywords:** Congenital, radioulnar, synostosis

## Abstract

Congenital radioulnar synostosis is a rare developmental skeletal malformation of the upper limb, characterized by the fusion of the proximal ends of the radius and ulna from birth. The failure of prenatal longitudinal segmentation of the adjacent radius and ulna results in a fibrous bony bridge between the radius and ulna. We present a 23-year-old female who presented with pain and restricted mobility of the left elbow joint for 7 years. A plain X-ray was performed for the patient, revealing a diagnosis of congenital radio-ulnar synostosis. Careful evaluation of the anatomical relations and spatial orientation of bony structures is required for the diagnosis and treatment of such cases.

## Introduction

Congenital radioulnar synostosis (CRUS) is an uncommon developmental skeletal malformation of the upper limb, characterized by a fusion of the proximal ends of the radius and ulna from birth [1]. In fact, the failure of prenatal longitudinal segmentation of the adjacent radius and ulna results in a fibrous bony bridge between radius and ulna [2]. Therefore, CRUS is thought to be caused by some in-utero insult. CRUS may result in severe disability especially when it is bilateral or there is considerable hyperpronation (i.e. ≥ 90) as adaptations are significantly poorer in such conditions [3]. Although the exact cause and pattern of inheritance of CRUS is still unknown, some studies have reported that it is inherited in an autosomal dominant manner [4]. Most often, congenital malformations are associated with chromosome X aberrations [5]. In 60% of cases, CRUS is bilateral and may be associated with other conditions such as hip dislocation, clubfeet, polydactyly or syndactyly and cardiac or urinary tract abnormalities [6].

Clinical features of CRUS include restricted movements of the forearm, especially rotation i.e. supination and pronation [7]. However, the condition is not usually painful until the subluxation of the radial head occurs [7]; hence the diagnosis of CRUS is usually delayed. Radiological examination reveals fusion or synostosis at the proximal ends of the radius and ulna [6]. Posterior dislocation of the radial head and proximal radioulnar fusion are other features of CRUS, which may coexist, in the same patient, as well as, it might present as separate entities [8]. The authors classify congenital radio-ulnar synostosis according to the degree of ossification, length of the synostosis and the involvement of the radial head [3]. Surgical management of CRUS is controversial and is based on individual functional limitations [7].

Mild deformity, minimal functional deficit and developed adjustments to activities are contraindications to surgical procedures [7]. Surgery is indicated in those who have bilateral CRUS or in those with severe rotational limitations [1]. Usually, surgery is recommended before the child starts schooling. The excision of the radial head at maturity is performed for symptomatic subluxation of the radial head [7]. Pronation of 15-60 degrees and over 60 degrees is relative and absolute indications for surgery, respectively [1]. To date, no case report has been published on CRUS from Saudi Arabia. Therefore, this case report is a valuable addition to the literature where we are presenting a 23-year old Saudi female with CRUS.

## Patient and observation

We are reporting the case of a 23-year-old married Saudi female patient, who presented to the King Khalid Hospital and Prince Sultan Centre for Health Services with pain and restricted mobility of the left elbow joint. The patient reported that this restriction in mobility significantly impacted her quality of life, in that she was unable to continue working as a seamstress or perform activities of daily living such as household chores or nursing her infant. Furthermore, she had experienced intermittent mechanical pain at the left elbow joint for the past 7 years, but the frequency and intensity of the pain had intensified ever since she started nursing her child 4 months ago. The character of the pain was reported to be dull, constant in nature and localized to the anterolateral aspect of the elbow joint. It was exaggerated upon movement and relieved upon rest. There was associated stiffness that lasted throughout the day. There was no radiation and no associated neurological symptoms such as numbness or weakness. The patient was not on any long-term medications and had minimal relief of her symptoms with oral paracetamol and topical NSAID (non-steroidal anti-inflammatory drug) ointment.

**Figure 1:**
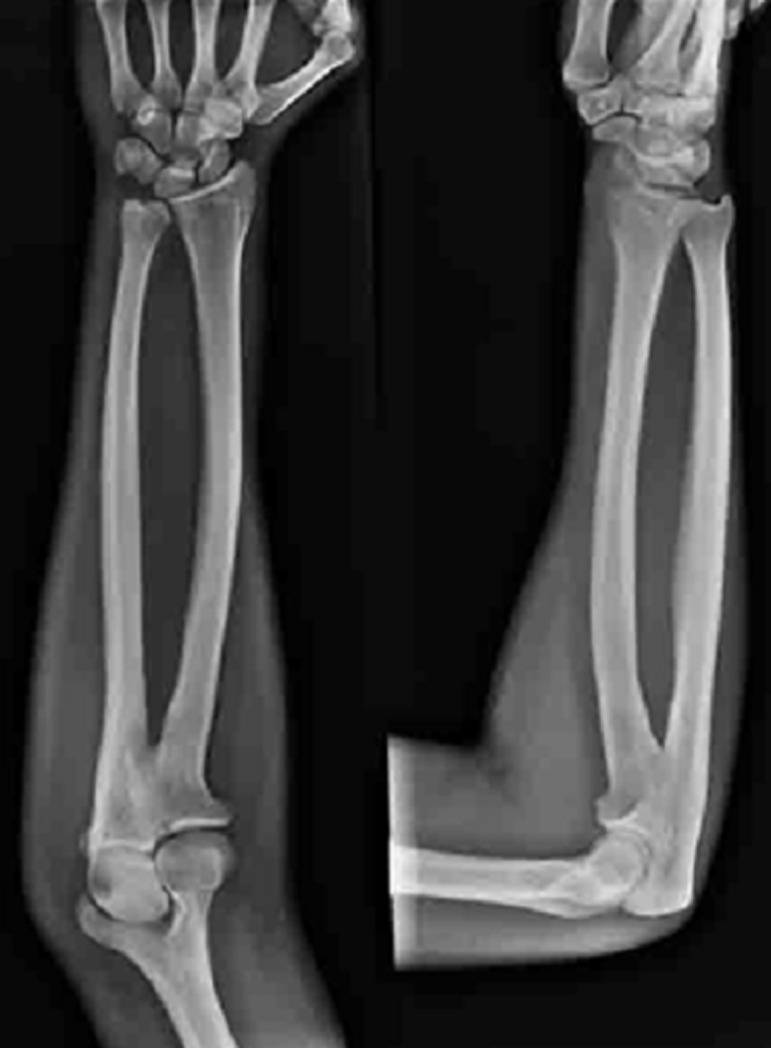
plain x-ray anterior and lateral views of the left elbow joint

**Figure 2:**
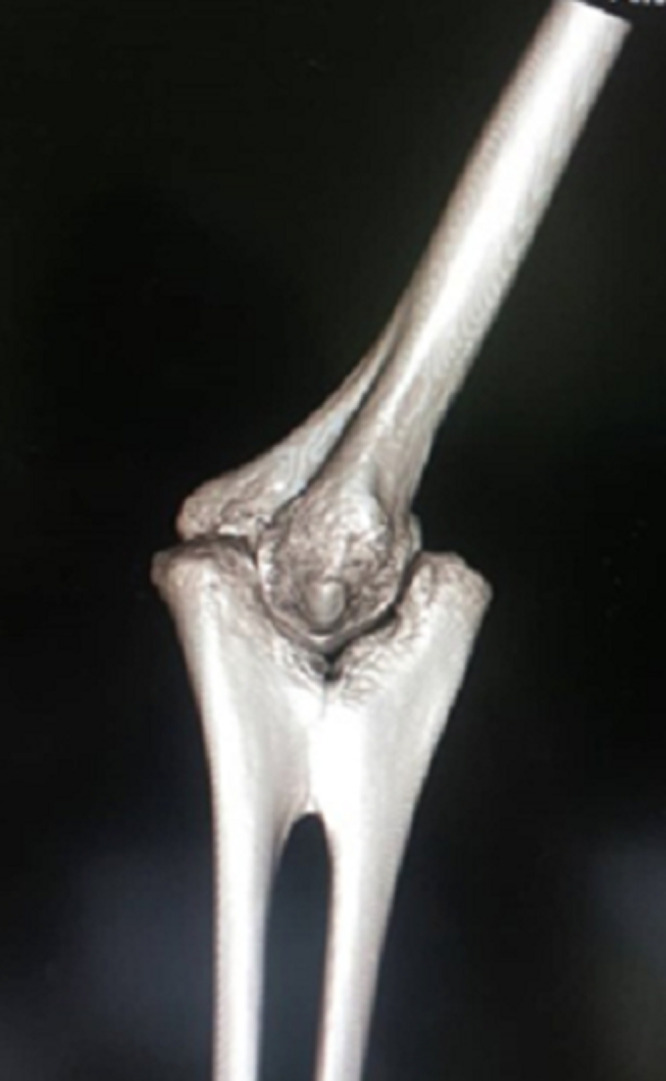
reconstructed image of left elbow joint (lateral view)

**Figure 3:**
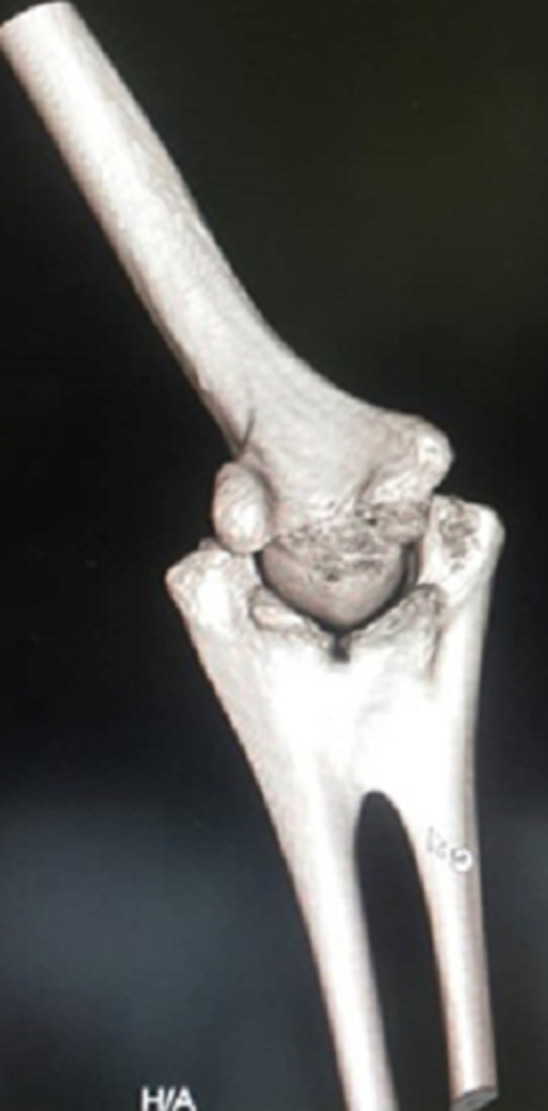
reconstructed image of left elbow joint (anterior view)

Neither positive family history of musculoskeletal deformities nor tobacco or alcohol consumption were performed. A review of her other systems revealed no gross abnormalities. On physical examination, there were no gross abnormalities of the overlying skin or elbow joint when inspected. Upon passive and active movement of the leftelbow joint, significant restriction in supination and pronation were observed. The patient informed that she was cognizant of this restriction since she was young. The neurovascular status of the limb was intact. A plain X-ray was performed for the patient, revealing a diagnosis of congenital radio-ulnar synostosis. The plain X-ray and reconstructed images are delineated in Figure 1, Figure 2 and Figure 3 respectively. Referencing Figure 1 below, the anterior and lateral views shows fusion of the proximal metaphysis of the left ulnar and radius. There is also a reduction of the radial head, shortening of the forearm and bowing of the radial shaft. The reconstructed images (Figure 2, Figure 3) corroborated these findings and the patient was diagnosed with a type 2; visible bony synostosis with a normal and reduced radial head.

## Discussion

At five weeks of gestation, the humours, radius and ulna are continuous with each other and joined together by a common perichondrium [9]. Then at the sixth week, tissues condensate to separate the cartilaginous anlage of the three bones. Congenital radio-ulnar synostosis results from the failure of the longitudinal separation and persistence of the cartilaginous anlage of the forearm during the 7^th^ week of gestation that results in a persistent bridge of tissue [4]. The persistent tissue bridge commonly ossifies into an osseous synostosis, but might also remain unossified as a fibrous synostosis [9]. It has also been reported to be associated with other congenital malformations such as hip dislocation, polydactyly and talipes equinovarus [5]. This patient was managed with non-steroidal anti-inflammatory drugs (NSAIDs), occupational therapy and activity modification. Like most other patients with congenital radio-ulnar synostosis, she was a poor candidate for surgery - it rarely succeeds in adult patients [6]. However, if diagnosed during childhood when it most frequently presents [1], derotational osteotomy is a viable surgical therapeutic strategy. Careful planning after a thorough evaluation of the anatomical relations and spatial orientation of bony structures is required [7].

## Conclusion

Careful thorough examination of upper limb at birth may offer some surgical correction of congenital radioulnar synostosis such as derotational osteotomy.
